# Low Prevalence of Pre-Treatment and Acquired Drug Resistance to Dolutegravir among Treatment Naïve Individuals Initiating on Tenofovir, Lamivudine and Dolutegravir in Zimbabwe

**DOI:** 10.3390/v15091882

**Published:** 2023-09-05

**Authors:** Vinie Kouamou, Tendai Washaya, Chiratidzo Ellen Ndhlovu, Justen Manasa

**Affiliations:** 1 Unit of Internal Medicine, Faculty of Medicine and Health Sciences, University of Zimbabwe, Harare P.O. Box A178, Zimbabwe; ratizw@gmail.com; 2 Biomedical Research and Training Institute, Harare P.O. Box A178, Zimbabwe; washayatendai@gmail.com (T.W.); jmanasa@gmail.com (J.M.)

**Keywords:** dolutegravir, HIV drug resistance, virological suppression, Zimbabwe, Africa

## Abstract

Dolutegravir (DTG) use in combination with tenofovir and lamivudine (TLD) is scaling up in Africa. However, HIV drug resistance (HIVDR) data to DTG remain scarce in Zimbabwe. We assessed the prevalence and genetic mechanisms of DTG resistance in people living with HIV initiating on TLD. A prospective cohort study was conducted between October 2021 and April 2023 among antiretroviral therapy (ART) naïve adults (≥18 years) attending care at an HIV clinic in Zimbabwe. Pre-treatment drug resistance (PDR) was assessed prior to TLD initiation and viral load (VL) outcome and acquired drug resistance (ADR) to TLD were described after 24 weeks follow-up. In total, 172 participants were enrolled in the study. The median (IQR) age and log_10_ VL were 39 (29–48) years and 5.41 (4.80–5.74) copies/mL, respectively. At baseline, no PDR to DTG was found. However, as previously reported, PDR to non-nucleotide reverse transcriptase inhibitor (NNRTI) was high (15%) whilst PDR to NRTI was low (4%). After a median duration of 27 (25–30) weeks on TLD, virological suppression (VL < 1000 copies/mL) was 98% and among the 2 participants with VL ≥ 1000 copies/mL, no ADR was found. HIVDR to DTG is rare among ART naïve individuals. DTG is more likely to address the problems of HIVDR in Africa.

## 1. Introduction

Despite the great achievements reported since the introduction of antiretroviral therapy (ART) and the efforts galvanized towards HIV/AIDS control and eradication by 2030, HIV drug resistance (HIVDR) may still pose an obstacle to the success of ART. Previously reported high levels of pre-treatment drug resistance (PDR) [1] to nucleotide reverse transcriptase inhibitors (NRTIs) and non-NRTIs (NNRTIs) prompted the World Health Organization (WHO) to recommend dolutegravir (DTG)-based regimens as the preferred first-, second- and third-line ART regimens for all people living with HIV (PLHIV) [2]. Reports from large clinical trials have well established that DTG, with its high potency and high genetic barrier to resistance, is likely to reduce the incidence of virological failure (VF) and the emergence of HIVDR among ART naïve individuals receiving DTG plus two NRTIs [3,4,5]. Furthermore, Ndashimye et al. (2021) showed that initiating patients on a DTG-containing regimen may encourage better clinical outcomes among ART naïve individuals in low- and middle-income countries (LMICs) [6]. In line with this, we recently reported high (95%) viral suppression (defined as a viral load greater or above 1000 copies/mL) among children and adolescents switching to a DTG-based regimen in rural Zimbabwe [7].

The WHO recommends that surveillance of drug resistance mutations (SDRMs) should accompany the scale-up of DTG-containing ART in HIV programmes and emphasizes the importance of estimating the extent to which acquired drug resistance (ADR) to DTG emerges in populations receiving DTG. Although cases of PLHIV developing VF and integrase strand transfer inhibitor (INSTI)-associated resistance mutation while on DTG-based regimens have been extremely rare [3,4,5], DTG is not impregnable to resistance [8,9] and may succumb to this phenomenon in many LMICs given its large scale-up and the limited viral load (VL) monitoring and genotypic resistance testing (GRT) [10].

Zimbabwe has adopted the use of DTG in combination with tenofovir and lamivudine (TLD) as the preferred first-line regimen for HIV treatment [11]. However, knowledge of major INSTI-DRMs and polymorphisms remains very limited in this geographical setting. Therefore, we assessed the prevalence and genetic mechanisms of DTG resistance among ART naïve individuals initiating DTG in a real-world setting.

## 2. Materials and Methods

### 2.1. Study Design, Setting and Population

This was a prospective cohort study investigating the prevalence and genetic mechanisms of DTG resistance among PLHIV in Zimbabwe. Participants were PLHIV initiating DTG-based ART between October 2021 and April 2023. Consenting participants were ART-naive adults (aged ≥18 years) initiating TLD or patients re-initiating first-line TLD after defaulting for at least 3 months. Participants were attending care at the Parirenyatwa Hospital Family Care Centre (PHFCC), a tertiary level setting, in Harare, Zimbabwe.

### 2.2. Participant Enrolment Procedure and Follow-up Visit

We consecutively invited individuals to participate in the study and obtained written informed consent prior to recruitment. Through an interview-based questionnaire, we extracted socio-demographic and clinical data (sex, age, marital status, occupation and education) as well as ART data (ART naïve or prior ART exposure). Upon enrolment, whole blood was collected in two ethylenediamine tetra acetic acid (EDTA) tubes; one tube for VL measurement and the other one for baseline GRT, respectively. These participants were followed up to or after 24 weeks when, besides routine laboratory tests, whole blood was collected for VL quantification and GRT for those with virological failure (VL ≥ 1000 copies/mL).

### 2.3. Viral Load Quantification 

Prior to HIV-1 VL quantification, the frozen plasma samples were allowed to thaw at room temperature for 30 min and then centrifuged. Plasma VLs were measured using the Xpert HIV-1 VL assay with a linear range of 40–10,000,000 HIV-1 ribonucleic acid (RNA) copies/mL at the Biomedical Research Training Institute (BRTI), Harare, Zimbabwe. The Xpert HIV-1 VL in vitro diagnostic assay is based on reverse transcriptase polymerase chain reaction (RT-PCR) technology. The Xpert HIV-1 VL automates the test process including RNA extraction, purification, reverse transcription and complementary deoxyribonucleic acid (cDNA) real-time quantitation in one fully integrated cartridge. Following the manufacturer’s instructions, in brief, 1000 μL of plasma sample was added to the cartridge, which was loaded into the instrument. This was followed by the automated process of nucleic acid purification and the simultaneous amplification and detection with the GeneXpert machine (Cepheid, Gauteng, South Africa). Some plasma samples were also measured at the Infectious Diseases Research Laboratory (IDRL), University of Zimbabwe with the Cobas Ampliprep/TaqMan48 HIV-1 Test quantification system, V2.0 (Roche, Indianapolis, IN, USA) with the detection limit of 20 copies/mL. Following the manufacturer’s instructions, in brief, 1050 μL of plasma was centrifuged and loaded onto the machine with the high and low positive controls and the negative control. This was followed by the automated process of extraction on the COBAS Ampliprep and real-time amplification and detection on the Taqman48 analyser. All tests were conducted in accordance with the manufacturer’s instructions.

### 2.4. Isolation of HIV Viral Ribonucleic Acid from Plasma Samples 

In preparation for HIV *pol* gene genotyping, HIV viral ribonucleic acid (RNA) was extracted from stored plasma samples using a column-based extraction kit, the QIAamp Viral RNA Mini kit protocol (Qiagen, Hilden, Germany). Following the manufacturer’s instructions, plasma samples were thawed, allowed to equilibrate to room temperature (15–25 °C) and thoroughly mixed by vortexing for 20 s. Lysis buffer AVL (560 μL) containing carrier RNA (1 ng/μL) was added to 140 μL of the plasma sample. The reaction was mixed by pulse-vortexing for 15 s and incubated at room temperature for 10 min. Absolute ethanol (560 μL at 96%) was added to the reaction and mixed by pulse-vortexing for 15 s in preparation for binding of the viral RNA onto the QIAamp mini-column membrane. The lysate obtained was transferred to a QIAamp mini-column membrane and centrifuged at 8000× *g* rpm for 1 min. The column was then washed twice with 500 μL each of buffer 1 and 2 respectively, followed by a centrifugation at full speed (15,000× *g* rpm) for 1 min in order to discard the flow through. Finally, 60 μL of elution buffer was added to the column and placed into a clean 1.5 mL microcentrifuge tube, which was incubated at room temperature for 1 min and centrifuged at 8000× *g* rpm for 1 min. The eluted viral RNA was immediately stored at −80 °C prior to RT-PCR. All tests were conducted in accordance with the manufacturer’s instructions.

### 2.5. Reverse Transcription, Amplification and Sequencing of the Extracted Viral RNA 

The extracted viral RNA was reverse-transcribed and amplified using the Applied Biosystems^TM^ TaqPath^TM^ Seq HIV-1 Genotyping Assay Kit (ThermoFisher Scientific, Waltham, MA, USA). The assay is a Sanger sequencing based-assay that enables the detection of genomic mutations in the protease (PR), reverse transcriptase (RT) and integrase (IN) regions of the HIV-1 *pol* gene. Briefly, for RT-PCR, 10 μL of RNA was denatured in a thermocycler for 10 min at 65 °C and added to a 40 μL Master reaction comprised of 39 μL of RT-PCR Master Mix, PR/RT or IN and 1 μL of enzyme (SuperScript^TM^ III One-Step RT-PCR with Platinum^TM^ Taq High Fidelity Enzyme). The mixture was then loaded onto the MIniamp thermal cycler (ThermoFisher Scientific, Waltham, MA, USA) under the following conditions: 1 cycle of 45 min at 50 °C for reverse transcription, 1 cycle of 2 min at 94 °C for enzyme inactivation, 40 cycles of 15 s, 20 s and 2 min at 94 °C, 50 °C and 72 °C for denaturation, annealing and extension, respectively and 1 cycle of 10 min at 72 °C for the final extension. Then, 2 μL of the resultant RT-PCR product was amplified immediately by nested PCR. For PR/RT and IN regions, nested PCR conditions were: 1 cycle of 4 min at 94 °C for enzyme inactivation, 40 cycles of 15 s, 20 s and 2 min at 94 °C, 53 °C and 72 °C for denaturation, annealing and extension, respectively, and 1 cycle of 10 min at 72 °C for the final extension. The resultant RT-PCR product was amplified immediately by nested PCR. All tests were conducted in accordance with the manufacturer’s instructions. The quality of the nested PCR product was assessed on a 1% agarose gel. The PR/RT and IN nested PCR product was purified using the Purelink Pro 96 viral RNA purification kit (Thermofisher Scientific, Waltham, MA,) per the manufacturer’s instructions. The purified product was sent to MCLAB, Molecular Cloning Lab, CA, USA for Sanger sequencing.

### 2.6. Bioinformatics Analysis of the Sequences

The AB1 files were exported from the instrument into a working folder on Geneious software version 11.0 [12], which allocated a percentage quality score (sequences with quality score >70% indicated a good sequence). Each of the 6 and 7 sequences for RT/PR and IN regions, respectively, were extracted by cutting off both ends of the sequences with bad quality. The sequences for each sample were mapped to a reference sequence to generate a consensus sequence. This consensus sequence (Fasta file) was exported for HIVDR classification and HIV-1 subtype identification on the online HIV Stanford database, Version 9.4.

### 2.7. Pre-Treatment Drug Resistance

Pre-treatment drug resistance was classified as surveillance drug-resistance mutations (SDRMs) using the calibrated population resistance (CPR) tool on the online Stanford HIV database. Genotypes were classified as wild type or SDRMs to NRTIs (M41L, K65R, D67NGE, T69Dins, K70RE, L74VI, V75MTAS, F77L, Y115F, F116Y, Q151M, M184VI, L210W, T215YFISCDVE, K219QENR); SDRMs to NNRTIs (L100I, K101EP, K103NS, V106MA, V179F, Y181CIV, Y188LHC, G190ASE, P225H, M230L); SDRMs to PIs (L23I, L24I, D30N, V32I, M46IL, I47VA, G48VM, I50VL, F53LY, I54VLMATS, G73STCA, L76V, V82ATFSCML, N83D, I84VAC, I85V, N88DS, L90M) and finally SDRMs to INSTIs (T66AIK, E92GQ, G118R, F121Y, E138AKT, G140ACS, Y143CHRS, S147G, Q148HRK, N155H, S230R and R263K) [13,14].

### 2.8. Acquired Drug Resistance Mutation

Acquired drug resistance was classified as the presence of any mutation that reduces susceptibility or virological response to tenofovir (K65R), lamivudine (M184V) and DTG (G118R, R263K and Q148HRK) as per the online Stanford HIV database.

### 2.9. Statistical Analysis

Statistical analyses were performed using Stata version 17.0 (StataCorp LP, College Station, TX, USA; 800-STATA-PC). Descriptive statistics were used to summarize the baseline demographic and clinical characteristics and were presented as proportions and medians (IQR). These characteristics were compared between ART-naive and prior ART-exposed participants using the Student’s *T*-test for parametric variables and the Mann–Whitney sum rank test for non-parametric variables. Fisher’s exact test was used for comparison of proportions of SDRMs between ART-naive and prior ART-exposed participants. Logistic regression was used to explore factors (age, baseline VL and CD4, ART history and sex) associated with any SDRM.

## 3. Results

### 3.1. Sociodemographic and Clinical Characteristics of All Participants

Between October 2021 and April 2023, a total of 172 participants were consecutively enrolled in the study. The median (IQR) age of the 172 participants was 39 (29–48) years, whereas the median (IQR) CD4 cell count and log_10_ VL were 175 (58–328) cells/mm^3^ and 5.41 (4.80–5.74) copies/mL, respectively. See Table 1. Slightly above half (54%) of the participants were females. The majority were married (54%) and had undergone a secondary education (78%). From these 172 participants,142 (83%) were ART naïve and 30 (17%) reported previous exposure to ART. Among those who had experienced ART, the majority, 43% (13/30), were on a DTG-based regimen as the previous ART. The proportion of ART defaulters was larger among males than females (67% vs. 33%, *p* = 0.012). Additionally, there were more divorced people among the ART defaulters than among the ART-naïve participants (27% vs. 10%, *p* < 0.001).

### 3.2. SDRMs among ART-Naïve and Prior ART-Exposed Participants

GRT of the *pol* (PR, RT and IN regions) gene was conducted on all the participants at baseline. Of the 172 participants, 137 (80%) and 150 (87%) were successfully genotyped for the PR/RT and the IN regions, respectively. The median (IQR) log_10_ VL of the 137 and 150 successfully genotyped was significantly higher than the 35 and 22 that failed genotyping, respectively [5.48 (5.03–5.83) vs. 4.60 (3.99–5.15) and 5.45 (4.92–5.80) vs. 4.50 (3.93–4.94), respectively with *p* < 0.0001]. All sequences were confirmed as HIV-1 subtype C.

**Figure 1:**
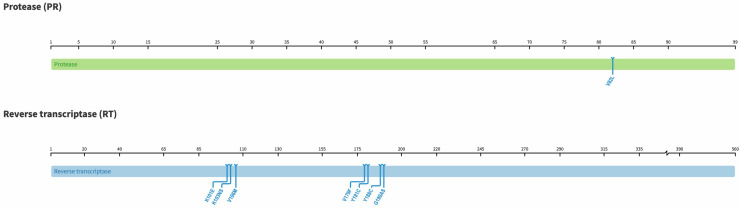
Diagram highlighting sites of mutations in the protease (99 amino acids) and reverse transcriptase (560 amino acids) regions of the HIV *pol* gene found in this study as per the HIV Stanford database, version 9.5.0.

The presence of any SDRM was seen in 19% (26/137). See Table 2. Although not statistically significant, these SDRMs were more seen among prior ART-exposed participants compared to ART-naïve participants (27% vs. 17%, *p* = 0.271, Fisher Exact test). SDRM to PI and NRTI was low, found in 1% (1/137) and 4% (5/137), respectively, whilst SDRMs to NNRTI was high, 15% (21/137). See Figure 1. SDRM to both NRTI and NNRTI (T69D + K103N) was found in one ART-naive participant. There were no SDRM to NRTI + NNRTI + PI. However, we found two PI accessory mutations (L10LF and Q58E) and 1 NRTI polymorphic mutation (S68G). The common NNRTI polymorphic accessory mutation E138A was found in 21 participants. E138A is found in persons receiving etravirine and rilpivirine and reduces their susceptibility by 2-fold.

There were no SDRMs to INSTI. However, four participants, all ART naïve, had INSTI accessory mutations (E157Q, Q95K, G163GR and L74LM+ T97A).

### 3.3. Factors Associated with the Presence of SDRMs

None of these factors, i.e., age, baseline VL and CD4, ART status and sex were associated with the presence of SDRMs among the participants (*p* > 0.05). See Table 3.

### 3.4. Acquired Drug Resistance at Follow-up Visit

Participants were followed up for a median (IQR) duration of 27 (25–30) weeks on TLD. Of the 131 participants with follow-up VL data available, the majority, 98% (129/131), were virologically suppressed with VL < 1000 copies/mL. The two participants with VL ≥ 1000 copies/mL did not have any SDRM at baseline and neither did they develop acquired drug resistance to DTG.

## 4. Discussion

The current WHO treatment guidelines for all PLHIV recommend the use of DTG-based regimens as the preferred first-line regimen because of its high potency and high genetic barrier to resistance. DTG plays a crucial role in the efforts to control the HIV pandemic in many resource-limited settings. In this study, we found no SDRM to DTG whilst finding a high level of SDRMs to NNRTI (15%) and low levels of SDRMs to NRTI (4%) and PI (1%) among ART-naïve participants initiating on TLD. Additionally, virological failure was low (2%), and no emergence of ADR to DTG was seen among the two failures at the follow-up visit.

The low prevalence of SDRMs to NRTI (4%) and PI (1%) along with the high prevalence of SDRMs to NNRTI (15%), particularly among ART defaulters is broadly consistent with our previous findings [15] and other available information on SDRMs to NNRTI [16,17,18]. Additionally, our results also confirm a previous report from the 2017 WHO survey on HIVDR that showed that PDR to NNRTIs had increased across all WHO regions [1]. Furthermore, our findings are in support of the current WHO guidelines on the use of DTG-based regimens as the preferred first-line so as to curb the problems of HIVDR and improve the rate of virological suppression in many LMICs.

We found that, by week 24, virological suppression VL < 1000 copies/mL and VL < 50 copies/mL on DTG was high (98% and 84%, respectively) among the participants. These findings were consistent with recent studies in LMICs that reported a high rate (>95%) of virological suppression among individuals on DTG [7,19]. Similarly, improved virological suppression in large clinical trials of ART-naïve individuals receiving DTG plus two NRTIs has been reported [20,21,22].

In the ADVANCE (South Africa) [20], NAMSAL (Cameroon) [21] and GEMINI-1 (multinational) [22] clinical trials, virological suppression was seen in 95%, 80% and 98%, respectively.

Unsurprisingly, prior to ART initiation, we found no SDRM to DTG among these ART-naïve and defaulted individuals initiating on TLD in a resource-limited setting (RLS). This is consistent with a previous study conducted in Zimbabwe which reported no SDRM to INSTI among INSTI-naïve individuals prior to the introduction of DTG [23]. Furthermore, in our study, among the participants with VL ≥ 1000 copies/mL at follow-up who underwent GRT, no emergent DRMs to DTG were found. Previous clinical trials studies have reported very low prevalence of emergent INSTI DRMs in ART-naïve individuals receiving DTG + two NRTIs. In the ADVANCE and NAMSAL clinical trials, no emergent INSTI DRMs were observed among PLHIV failing their treatment [3,5,20,21]. However, INSTI DRMs were reported after a longer period (by week 48 and 96) than this current study (by week 24). In the STAT clinical trial, no emergent INSTI-associated DRMs were reported by week 24 and 48 among ART-naïve PLHIV receiving DTG plus 3TC [24,25].

Additionally, the recent OPTIPRIM2-ANRS 169 and the Gilead’s phase 2 trials (NCT02397694) have reported no emergent INSTI resistance among ART-naïve individuals failing on DTG [26,27]. In these two studies, INSTI DRMs were reported by week 48. All these findings are in support of the high potency and high genetic barrier to resistance associated with the use of DTG [28].

Lastly, in our study, up to 17% of the participants were individuals who had previously defaulted ART. Just under half, or 43% (13/30), of these participants were on a DTG-based regimen as previous ART. Previous studies have shown that ART defaulters had higher levels of PDR to NNRTI which may predispose them to virological failure and further development of HIVDR once on ART [1,15]. In our study, we did not observe this phenomenon with DTG. Nevertheless, strategies to retain people on ART in many RLS should be reinforced as these people may likely present for care with advanced HIV disease, increasing their risk to HIV morbidity and mortality, as well as with further emergent HIVDR.

Overall, this study has provided baseline SDRMs to the preferred first-line ART (TLD) in a RLS and assessed the extent to which acquired DTG resistance emerges in populations receiving DTG. One limitation of the study is the small sample size. Another limitation is the short duration of follow-up. However, previous studies with longer follow-up duration did not find any emergent INSTI DRMs among ART-naïve individuals failing their treatment.

## 5. Conclusions

We found no SDRM and acquired drug resistance to DTG among ART-naïve individuals initiating TLD in a RLS following 6-month post-DTG initiation. DTG is more likely to address the problems of virological failure and emergence of HIVDR in Africa. However, as DTG becomes widely available in many RLS, surveillance of HIVDR to DTG among individuals on DTG is warranted so as to preserve its activity.

## Figures and Tables

**Table 1:** Sociodemographic and clinical characteristics of all the participants.

Characteristics	All Participants n = 172	ART Naïve n = 142	ART Defaulted n = 30	*p* Value
Age, years, median (IQR)	39 (29–48)	40 (31–49)	37 (28–44)	0.114
Sex, n(%) Female Male	93 (54%) 79 (46%)	83 (58%) 59 (42%)	10 (33%) 20 (67%)	**0.012**
Plasma VL in log_10_ copies/mL, median (IQR)	5.41 (4.80–5.74)	5.42 (4.78–5.76)	5.32 (4.80–5.74)	0.637
CD4 cell count in cells/mm^3^, median (IQR)	175 (58–328)	165 (55–357)	213 (77–320)	0.795
Marital status, n(%) Married Divorced Single Widowed	93 (54%) 23 (13%) 41 (24%) 15 (9%)	86 (61%) 15 (10%) 28 (20%) 13 (9%)	7 (23%) 8 (27%) 13 (43%) 2 (7%)	**<0.001**
Education, n(%) None Primary Secondary Tertiary	1 (1%) 14 (8%) 135 (78%) 22 (13%)	1 (1%) 11 (8%) 111 (78%) 19 (13%)	0 (0%) 3 (10%) 24 (80%) 3 (10%)	0.847
Occupation, n(%) Employed Unemployed Student	79 (46%) 89 (52%) 4 (2%)	69 (48.5%) 69 (48.5%) 4 (3%)	10 (33%) 20 (67%) 0 (0%)	0.364
Previous ART, n(%) ABC/3TC/ATV/r ABC/3TC/DTG AZT/3TC/ATV/r TDF/3TC/ATV/r TDF/3TC/DTG TDF/3TC/EFV		0 0 0 0 0 0	1 (3.3%) 2 (6.7%) 2 (6.7%) 2 (6.7%) 11 (36.6%) 12 (40%)	NA

IQR = Interquartile range, VL = Viral load, ART = Antiretroviral therapy, ABC = Abacavir, 3TC = Lamivudine, ATV/r = Atazanavir/ritonavir, DTG = Dolutegravir, AZT = Zidovudine, EFV = Efavirenz, TDF = Tenofovir disoproxil fumarate, NA = Not applicable.

**Table 2:** Surveillance drug resistance mutations among the participants.

Sequence IDs	ART Status	NRTI SDRMs	NNRTI SDRMs	PI SDRMs	INSTI SDRMS
PDR_028	ART naive	None	K103N, V106M	None	None
PDR_030	ART naive	None	K103N	None	None
PDR_032	ART defaulted	None	K103N	None	None
PDR_038	ART naive	None	K103N	None	None
PDR_039	ART naive	D67E	None	None	None
PDR_040	ART naive	None	V179F	None	None
PDR_041	ART naive	None	K103N	None	None
PDR_057	ART defaulted	None	K101E, G190S	None	None
PDR_064	ART naive	None	K103N	None	None
PDR_067	ART naive	None	K103N	None	None
PDR_068	ART defaulted	M184I	None	None	None
PDR_072	ART naive	D67G	None	None	None
PDR_077	ART naive	None	K103N	None	None
PDR_080	ART naive	None	K101E	None	None
PDR_090	ART naive	None	K103N	None	None
PDR_092	ART naive	None	K103N, Y181C	None	None
PDR_117	ART defaulted	None	K103N, Y188C	None	None
PDR_122	ART naive	None	K103N, V179F	None	None
PDR_127	ART defaulted	None	K103N, Y181C, G190A	None	None
PDR_132	ART defaulted	None	K101E, G190A	None	None
PDR_140	ART naive	K219R	None	None	None
PDR_14	ART defaulted	None	K103NS	None	None
PDR_155	ART naive	T69D	K103N	None	None
PDR_174	ART naive	None	K103N	None	None
PDR_175	ART naive	None	K103N	None	None
PDR_179	ART naive	None	None	V82L	None

NRTI = Nucleotide reverse transcriptase inhibitors, NNRTI = Non-NRTI, PI = Protease inhibitor, INSTI = Integrase strand transfer inhibitor, SDRMs = Surveillance drug resistance mutations.

**Table 3:** Factors associated with the presence of SDRMs among the participants.

Characteristics, n = 172	Univariate Analysis			
	Proportion	OR	95% CI	*p* Value
Age, years <40 ≥40	0.51 0.49	0.49	0.20–1.16	0.106
Baseline CD4, cells/mm^3^ <200 ≥200	0.53 0.47	0.77	0.32–1.85	0.556
Baseline VL, copies/mL <100,000 ≥100,000	0.32 0.68	2.23	0.79–6.28	0.128
ART status ART naïve ART defaulted	0.83 0.17	1.97	0.74–5.22	0.172
Sex Male Female	0.46 0.54	0.83	0.36–1.90	0.652

ART = Antiretroviral therapy, OR = Odds ratio, CI = Confidence interval, VL = Viral load.

## Data Availability

Sequence data were submitted to GenBank, and accession numbers will be provided at a later stage.
